# Skills to Perform Vessel Eversion in Mouse Cervical Cardiac Transplantation with Cuff Technique

**DOI:** 10.21470/1678-9741-2020-0125

**Published:** 2021

**Authors:** Liang Tan, Xubiao Xie, Yanan Xu, Qianchuan Tian, Qian Zhang, Gongbin Lan, Hongxia Wang, Yong Zhao, Longkai Peng

**Affiliations:** 1 Department of Kidney Transplantation, The Second Xiangya Hospital of Central South University, Changsha, Hunan, China.; 2 Clinical Research Center for Organ Transplantation in Hunan Province, The Second Xiangya Hospital of Central South University, Changsha, Hunan, China.; 3 Transplantation Biology Research Division, State Key Laboratory of Membrane Biology, Institute of Zoology, Chinese Academy of Sciences, Chaoyang District, Beijing, China.; 4 Laboratory Medicine Center, Nanfang Hospital, Southern Medical University, Guangzhou, Guangdong, China.

**Keywords:** Heart Transplantation, Tissue Donors, Heart, Vascular Factor, Surgical Instruments, Animals

## Abstract

**Introduction:**

The mouse heterotopic cardiac transplant model has been extensively used to explore transplant immunity. Although the cuff technique facilitates the operation, the procedure remains difficult, and vessel eversion is the most difficult step. Cuff movement and everted vessel wall slippage are the main adverse factors in vessel eversion. Traditional strategies to prevent these factors focus on cuff fixation, while more steps or surgical instruments would be required.

**Methods:**

According to the reported protocols and our experience, the vessel eversion skills were modified and used for transplantation. Cardiac grafts from C57BL/6(H-2^b^) or BALB/c(H-2^d^) mice were transplanted into C57BL/6(H-2^b^) mice. The operating times of recent 90 operations, which were divided into 9 groups according to their sequence, were summarized and analyzed.

**Results:**

The mouse cervical cardiac transplantation was successfully performed by using the modified vessel eversion skills. The cuff movement, which is the most important adverse factor to prevent vessel eversion, was effectively prevented. In the recent 90 operations, the total operating time was 47.3±7.9 min and the success rate was 98%.

**Conclusions:**

The modified surgical skills simplify the vessel eversion in mouse cervical cardiac transplantation with cuff technique, characterized by less cuff movement, fewer steps, and surgical instruments. Using these surgical skills, the transplant can be performed in a short time.

**Table:** 

Abbreviations, acronyms & symbols	
**CA**	**= Carotid artery**
**EJV**	**= External jugular vein**

## INTRODUCTION

Corry first described the mouse heterotopic vascularized cardiac transplant model in 1973^[1]^, and Chen first described mouse cervical cardiac transplantation in 1991^[2]^. In this nonfunctional transplant model, the cardiac graft aorta is anastomosed to the recipient artery, and the cardiac graft pulmonary artery is anastomosed to the recipient vein; the host blood flow perfuses the cardiac graft through the coronary circulation. Although the mouse heterotopic cardiac transplant model has been extensively used to explore transplant immunity^[3,4]^, the procedure remains difficult. To simplify the operation, cuff technique was developed for mouse cervical cardiac transplantation in 1991^[5]^.

Although the cuff technique facilitates the mouse cervical cardiac transplantation, the procedure is still difficult. During the surgery, vessel eversion is the most difficult step; cuff movement and everted vessel wall slippage are the main adverse factors in vessel eversion^[3,6-8]^. Traditionally, strategies to prevent these problems have focused on cuff fixation, such as the use of a specially designed cuff that has an extension, or on modification of the manner of vessel anastomosis or the use of affiliating tools^[3,6-8]^, which required more steps or surgical instruments. In this article, we present surgical skills to simplify vessel eversion that do not require specially designed cuffs or surgical instruments.

## METHODS

### Animals

The 6-12-week-old male C57BL/6(H-2^b^) and BALB/c(H-2^d^) mice were purchased from Beijing HFK Bioscience Co., Ltd, or Beijing SPF Biotechnology Co., Ltd, or generated by the Institute of Zoology. All mice were kept in microisolator cages in a specific pathogen-free facility. All experiments were approved by the Animal Ethics Committee of the Institute of Zoology. All animals received human care in compliance with the Guide for the Care and Use of Laboratory Animals.

### Surgical Instruments and Materials

**Fig. 1:**
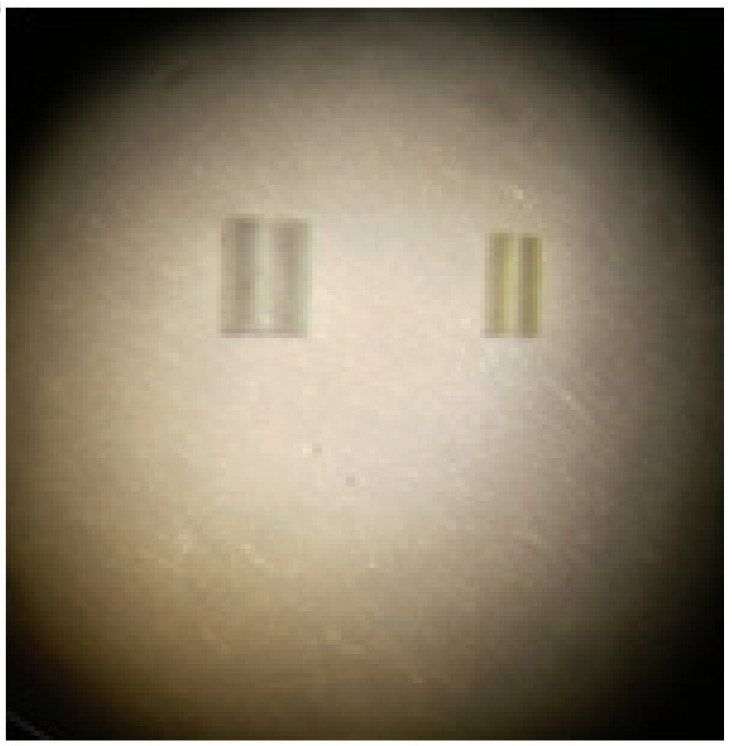
Cuffs. The left cuff is for external jugular vein (EJV) eversion, and the right cuff is for carotid artery (CA) eversion.

The cuffs were designed in two sizes. The cuff for the external jugular vein (EJV) has a 0.8 mm external diameter and a 0.7 mm inner diameter. The cuff for the carotid artery (CA) has a 0.5 mm external diameter and a 0.4 mm inner diameter. The cuffs were cut in segments ranging from 1 to 1.3 mm (Figure 1). A set of microsurgical instruments included two curved forceps with a 0.15 mm tip for tissue isolation and vessel eversion, and two curved forceps with a 0.30 mm tip for knotting. Microvascular clamps were purchased from Scanlan (Ref. 1001-531). Braintree Scientific, Inc. (Ref. 103-S) supplied the 7-0 silk.

### Microscope

The operation was conducted using an operating microscope (Luckbird, Zhenjiang Zhongtian Optical Instruments Co., Ltd., Zhenjiang, China) with a magnification between 5x and 25x.

### Cardiac Graft Procurement and Transplantation

**Fig. 2:**
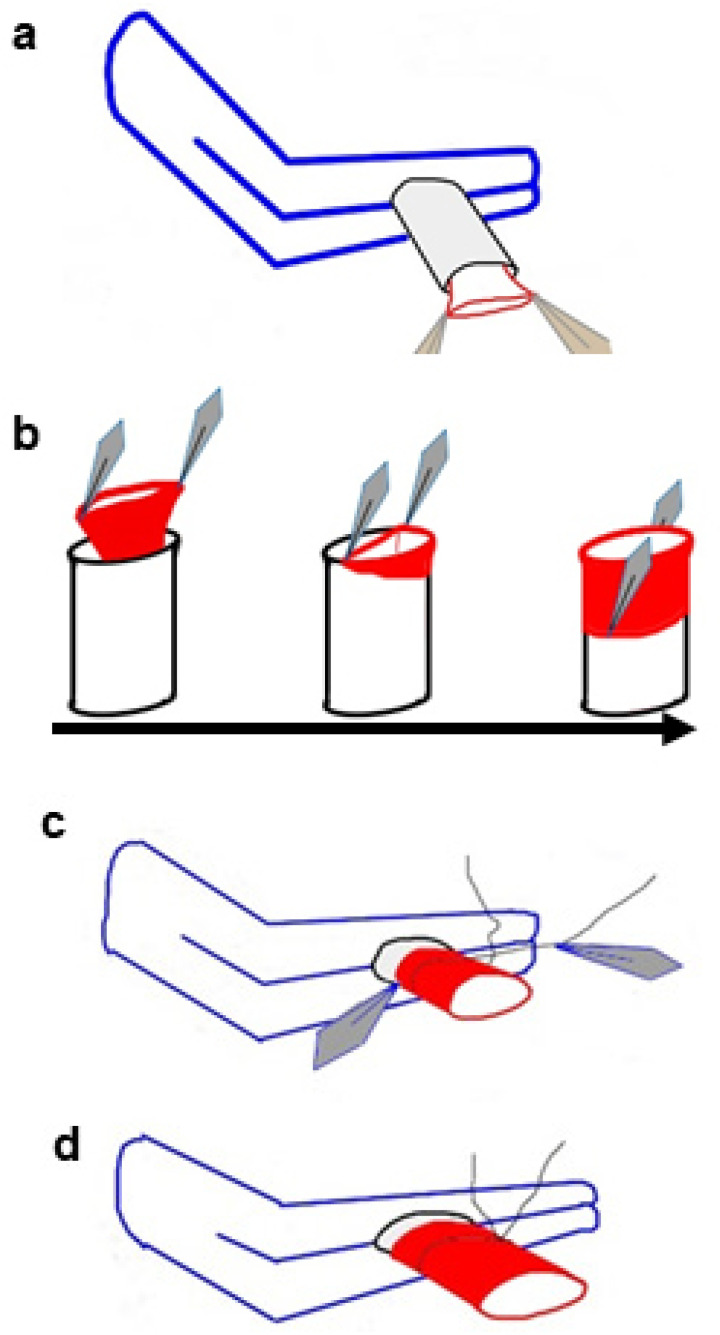
Diagram of vessel eversion. (A) The vessel is dragged into the cuff, and the vessel end is gently caught with two forceps bilaterally, while the cuff is perpendicular and clamped. (B) First, half of the vessel end is everted over the cuff, followed by the other half of the vessel end eversion. (C) A preset circumferential ligature encircles the everted vessel wall, and one end of the ligature is gently pulled to prevent slippage of the everted vessel wall. (D) The ligature is tied, and the vessel eversion is finished. The gray diamond represents the tip of the forceps; red represents the vessel wall.

The cardiac graft procurement and transplantation primarily followed the reported protocols^[5,9,10]^, modified by our experience. In the step of recipient vessel eversion, the vessel was dragged into the cuff, and the vessel end was gently caught with two forceps, bilaterally, while the cuff was perpendicular to the clamp (Figure 2A). One half of the vessel end was everted over the cuff first, followed by the other half of the vessel end eversion (Figure 2B). To connect the everted vessel wall to the cuff, the surgeon held the vessel wall with one forceps, caught one end of a preset circumferential ligature with the other forceps, and encircled the everted vessel wall with this ligature. Then, the surgeon gently fixed the end of the ligature to the everted vessel wall and released the first forceps to tie the ligature (Figures 2C and 2D). A single surgeon finished the cardiac graft procurement and transplantation. Transplant rejection was defined as cardiac graft arrest.

### Statistical Analysis

All data were presented as mean±SEM. Graft survival was compared using the log-rank test. A *P* <0.05 was considered statistically significant.

## RESULTS

The Mouse Cervical Cardiac Transplantation Model with Cuff Technique Was Successfully Established

**Fig. 3:**
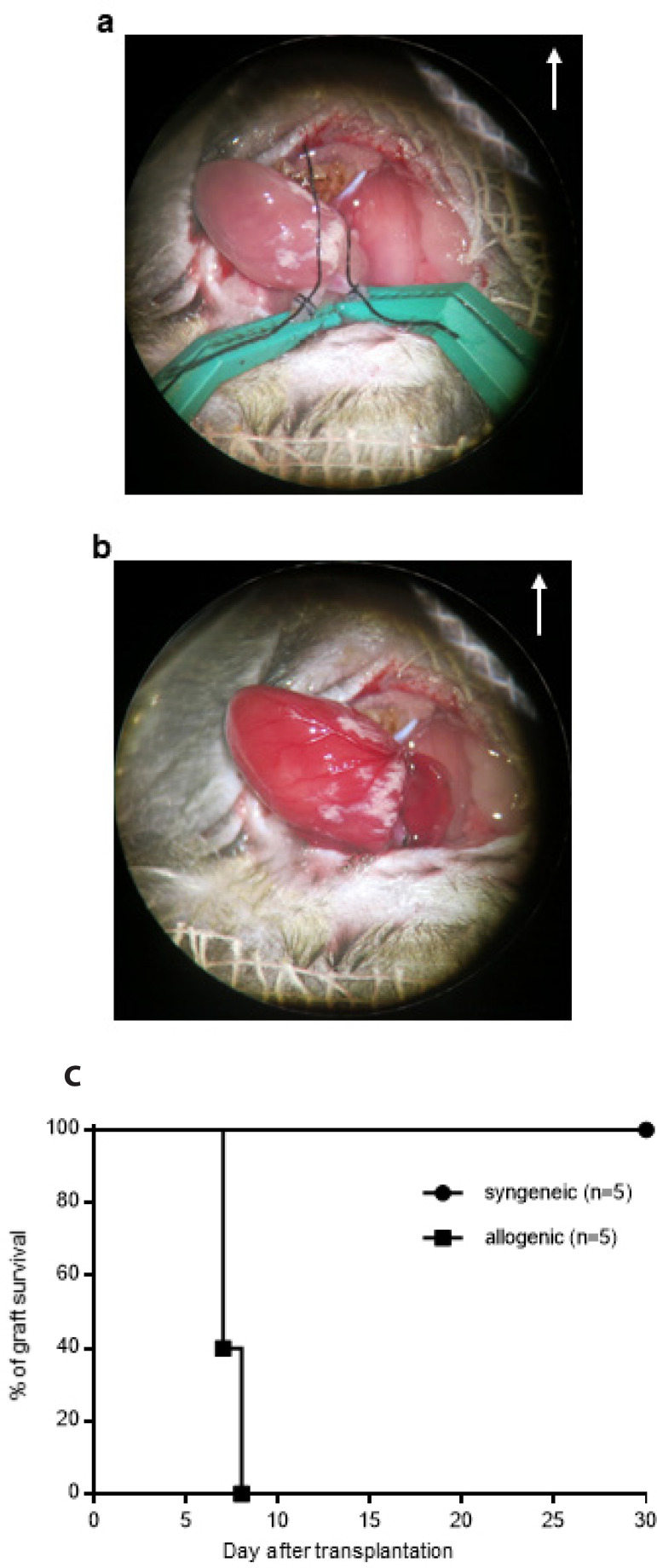
Mouse cervical cardiac transplantation model with a cuff technique is established. (A) The cardiac graft is implanted. (B) Recipient blood perfuses the cardiac graft. (C) Survival curve of the syngeneic or allogeneic grafts. The white arrow points toward the head of the recipient mouse.

According to the reported protocols^[5,9,10]^ and our modification during training, the mouse cervical cardiac transplantation was established successively (Figures 3A and 3B). The survival time of allogeneic grafts was 7 to 8 days, and syngeneic grafts had a long-term survival rate (P<0.005) (Figure 3C). In the 90 most recent operations, only 2 transplantations failed; the cause was vascular torsion after cardiac graft shift.

### Vessel Eversion Can Be Successfully Performed in a Short Time

**Fig. 4:**
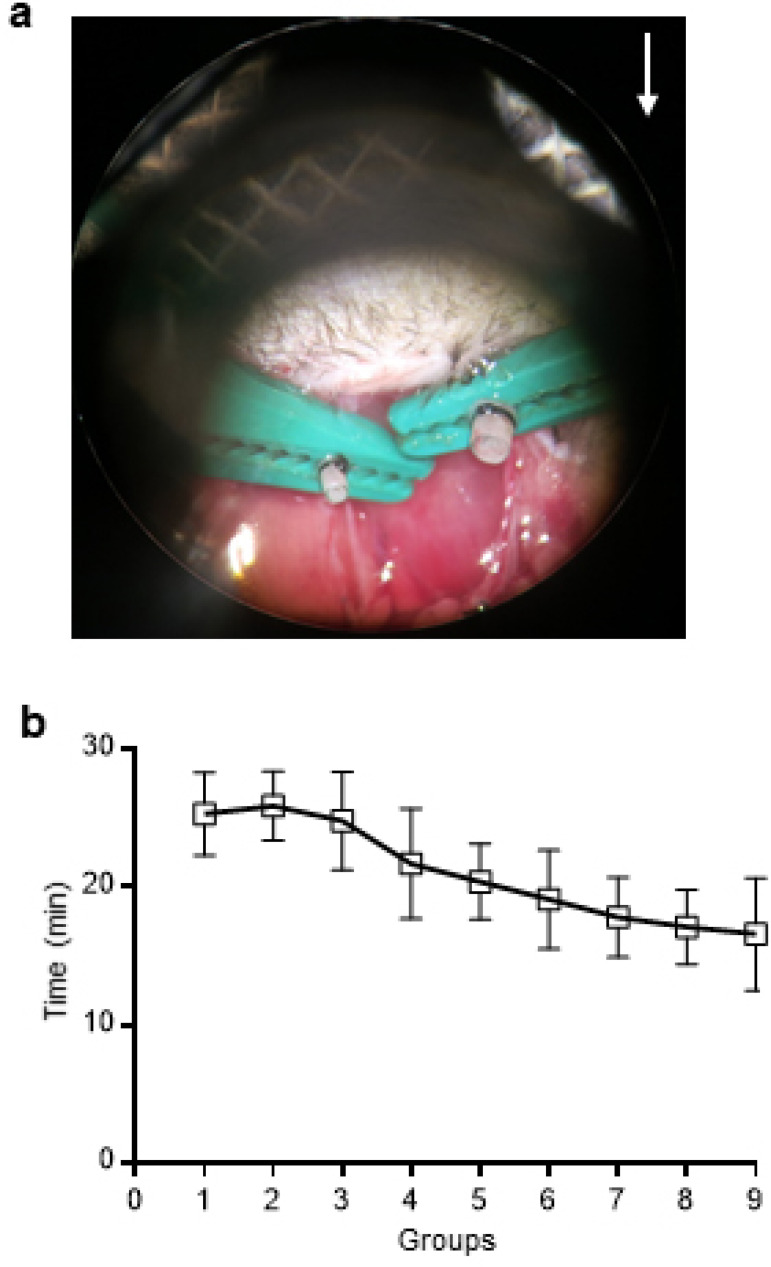
Vessel eversion can be successfully performed in a short time. (A) Both EJV and CA of the recipient mouse were everted. (B) The vessel preparation time was short. White arrow points toward the head of the recipient mouse.

Vessel eversion is the most difficult step of mouse cervical cardiac transplantation using the cuff technique. Cuff movement and everted vessel wall slippage are the main adverse factors of vessel eversion^[3,6-8]^. Following the surgical skills previously described, vessel eversion can be successfully performed (Figure 4A). Vessel preparation, which extends from performing recipient skin incision to finishing the vessel eversion of both EJV and CA, can be performed in a short time. In 90 recent operations, the time required for vessel preparation was 21.0±4.8 min. The 90 operations were divided into 9 groups according to their time sequence; the time of vessel preparation was stable with a declining trend (Figure 4B). Therefore, the surgical skills are effective to perform the vessel eversion.

### The Transplant Can Be Finished in A Short Time

**Fig. 5:**
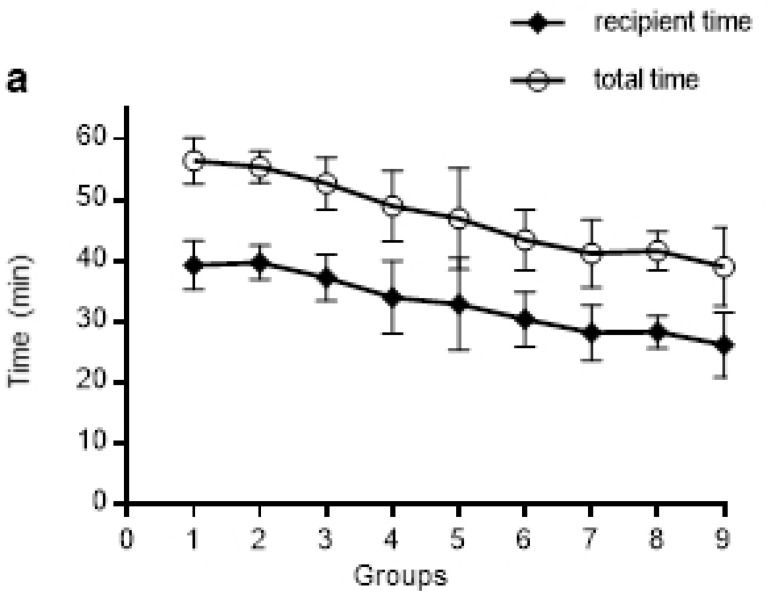
Transplantation can be performed in a short time. The total time required for transplantation and the recipient time were short. The recipient time included time of vessel preparation, graft implantation, and recipient incision suture. ***, P <0.005.

Due to the benefit of vessel eversion surgical skills, the transplant can be completed in a short time. In our recent 90 consecutive operations, the total time was 47.3±7.9 min from the beginning of the graft procurement to the incision suture of the recipient mice. Furtherly, these 90 operations were divided into 9 groups according to their time sequence. In the last 10 operations, the operating time was stably decreased and totaling 39.0±6.4 min. The recipient time, including vessel preparation, graft implantation, and recipient incision suture, also decreased (Figure 5). Therefore, improved operational skills significantly shortened the operating time.

## DISCUSSION

The mouse heterotopic cardiac transplant model has been extensively used to explore transplant immunity^[3,4]^. Compared to the suture anastomosis, deploying a cuff technique may be easier and saves time^[5,11]^. The most difficult step of mouse cervical cardiac transplantation using the cuff technique is vessel eversion, especially for the CA^[8,10]^. Cuff movement and everted vessel wall slippage are the primary adverse factors in vessel eversion^[3,6-8]^. Some work has been done to address these adverse factors, such as using a cuff with an extension that can be held with a clamp and using instruments to hold the cuffs. These improvements help make vessel eversion easier in situations requiring more steps or surgical instruments. In addition, improving surgical skills may be another way to facilitate vessel eversion.

During our operations, we first placed the cuff perpendicular to the clamp and then everted the vessel wall half in half. To prevent vessel wall slippage during ligation after eversion, one forceps held the vessel wall while the other forceps caught one end of a preset circumferential ligature. The second forceps encircled the everted vessel wall with this ligature, then gently pulled the end of the ligature to fix the everted vessel wall. The surgeon released the first forceps to tie the ligature. In our experience, the adverse effects of cuff movement and everted vessel wall slippage can be prevented using these surgical skills.

Traditionally, the average total operating time has been reported to be 45 to 60 min^[9-12]^; the total operating time of our recent 90 consecutive operations was 47.3±7.9 min. In addition to the total time, the time of vessel preparation, defined as the process from performing the recipient skin incision to finishing the vessel eversion of both EJV and CA, was 21.0±4.8 min for these 90 operations. Our improvement in surgical skills was effective in reducing the time of operations.

Several useful points and some troubleshooting are as follows:

Watching a video of the operation is helpful for mastering the operation. Some videos by Ratschiller and Oberhuber are available online^[10,12,13]^.

Super-fine-tip forceps, such as the forceps with a 0.15 mm tip, are more appropriate during blood vessel isolation, eversion, and anastomosis.

An inner core, which is placed into the vessel lumen to facilitate vessel eversion, may be useful during the early training stage. Our experience, however, indicates that it should be avoided as much as possible because of the risk of vessel intima injury and thrombosis for some genetically modified mice.

The site of the ligature on the recipient CA must be at the proximal site of its bifurcation, and some distance should be left from the ligature to the bifurcation to avoid the risk of cerebral blood flow disorder.

The length of the cuffs should be appropriate for vessel eversion and donor and recipient vessel anastomosis. In our experience, 1.0-1.3 mm may be suitable.

In conclusion, in addition to the strategies that facilitate vessel eversion by fixing the cuff in mouse cervical cardiac transplantation with cuff technique, the modified surgical skills simplify the vessel eversion, characterized by less cuff movement, fewer steps and surgical instruments. Using these surgical skills, the transplantation could be performed in a short time.

**Table:** 

Authors' roles & responsibilities	
LT	Substantial contributions to the conception or design of the work; or the acquisition, analysis or interpretation of data for the work
XX	Substantial contributions to the conception or design of the work; or the acquisition, analysis or interpretation of data for the work
YX	Substantial contributions to the conception or design of the work; or the acquisition, analysis or interpretation of data for the work
QT	Substantial contributions to the conception or design of the work; or the acquisition, analysis or interpretation of data for the work
QZ	Substantial contributions to the conception or design of the work; or the acquisition, analysis or interpretation of data for the work; final approval of the version to be published
GL	Substantial contributions to the conception or design of the work; or the acquisition, analysis or interpretation of data for the work; final approval of the version to be published
HW	Substantial contributions to the conception or design of the work; or the acquisition, analysis or interpretation of data for the work; drafting the work or revising it critically for important intellectual content; final approval of the version to be published
YZ	Substantial contributions to the conception or design of the work; or the acquisition, analysis or interpretation of data for the work; drafting the work or revising it critically for important intellectual content; final approval of the version to be published
LP	Substantial contributions to the conception or design of the work; or the acquisition, analysis or interpretation of data for the work; final approval of the version to be published
